# Does rumination mediate the relationship between attentional control and symptoms of depression?

**DOI:** 10.1016/j.jbtep.2018.12.007

**Published:** 2019-06

**Authors:** Hannah DeJong, Elaine Fox, Alan Stein

**Affiliations:** a Department of Psychiatry, University of Oxford, Warneford Hospital, Oxford, OX3 7JX, UK; b Department of Experimental Psychology, University of Oxford, New Radcliffe House, Radcliffe Observatory Quarter, 49 Walton Street, OX2 6AE, Oxford, UK

**Keywords:** Attentional control, Depression, Rumination, Emotional regulation

## Abstract

**Background and objectives:**

It has been suggested that impaired attentional control (AC) promotes the use of maladaptive emotional regulation strategies, such as rumination, with subsequent increase in risk of depression.

**Method:**

This study examined this hypothesis in a healthy community sample. Questionnaire measures of depression, anxiety, rumination and self-reported AC (shifting and focusing) were used, as well as an attention performance task (Attention Network Task; ANT).

**Results:**

While self-report and performance measures of AC were not significantly related, both depression and rumination were associated with reduced self-reported AC. Depression was specifically associated with poorer attentional shifting. Depression and brooding were also associated with *better* performance on the conflict component of the ANT. Importantly, the relationships of ANT conflict and self-reported AC to depression were mediated by brooding.

**Limitations:**

The current study used a community sample, and it is unclear if results would generalise to a clinical population. All measures were taken concurrently and so it is not possible to confidently ascertain causality or direction of effects.

**Conclusions:**

These results are consistent with the suggestion that impaired AC, particularly a narrow and inflexible attentional focus, may increase risk of depression by promoting ruminative thinking. The results highlight the importance of considering both self-report and performance measures of AC, as well as different components of attentional performance.

## Highlights

• Poor attentional control (AC) may increase depression risk, via increased rumination.
• Both performance and self-report measures of AC were examined.
• Depression and rumination were associated with poorer self-reported AC.
• Depression and rumination were associated with better conflict performance.
• Relationships between AC and depression were mediated by brooding rumination.

## 1 Introduction

Attentional control (AC) refers to the ability to direct and focus attention, and is considered to be an important component of executive functioning and cognitive control (Derryberry & Reed, 2002; Eysenck, Derakshan, Santos, & Calvo, 2007). AC has clear implications for cognition, learning and daily functioning, but also for emotional regulation and the development of psychological disorder. It has been suggested, for instance, that poor AC is associated with impairments in emotional regulation (Joormann & D'Avanzato, 2010; Joormann & Vanderlind, 2014; Ernst H. W.; Koster, De Lissnyder, Derakshan, & De Raedt, 2011), which may then confer risk for developing affective disorder. In this study, we explore the links between AC, rumination and symptoms of depression.

### 1.1 Measuring attention functioning

Attention performance has been measured using both performance and self report measures. We focus in this study on two commonly used measures: firstly, the Attention Network Task (ANT) (Fan, McCandliss, Sommer, Raz, & Posner, 2002) and secondly, the Attentional Control Scale (ACS) (Derryberry & Reed, 2002). The ANT is based on Posner and colleagues' model of attention (Posner & Petersen, 1990; Posner & Rothbart, 2007), which proposes that attention functioning can be divided into three distinct but interrelated networks: alerting, orienting and executive attention. Alerting is involved in maintaining sensitivity to incoming information. Orienting is implicated in directing attention to relevant information in the environment. Executive attention is primarily involved in resolving conflicts between competing information. The ANT is designed to assess the functioning of all three networks in a single task.The Attentional Control Scale (ACS) (Derryberry & Reed, 2002) is a self-report questionnaire measure, with items framed in terms of everyday functioning, e.g. ‘It's hard for me to concentrate on a difficult task when there are noises around’. Factor analyses have indicated that the ACS contains two components: focusing and shifting (Judah, Grant, Mills, & Lechner, 2014; Ólafsson et al., 2011). Focusing is defined as the ability to maintain attention, particularly in the face of distraction or competing stimuli. Shifting involves the flexible allocation of attention, and the ability to move attentional resources between different tasks or sources of information.

The relationship between self-reported measures of AC and performance measures has been addressed in relatively few studies, with mixed findings (Reinholdt-Dunne, Mogg, & Bradley, 2009, 2013; Judah et al., 2014; Pacheco-Unguetti, Acosta, Marqués, & Lupiáñez, 2011; Tortella-Feliu et al., 2014; Williams, Rau, Suchy, Thorgusen, & Smith, 2017). It therefore remains somewhat unclear how these are related and whether this differs across different populations. Comparisons between self-reported attention and the ANT specifically have either shown no significant correlation between these measures (Reinholdt-Dunne, Mogg, & Bradley, 2009; Williams et al., 2017), or correlations only between focusing and the conflict index of the ANT (Reinholdt-Dunne et al., 2013). Notably, many of the existing studies have examined only total ACS scores, and have not considered possible differences between the focusing and shifting components. Distinguishing between these subscales may help to clarify some of the inconsistent findings in this area.

### 1.2 Attentional control and depression

Poor AC is significantly associated with vulnerability to affective disorder. Self-reported difficulties with AC are associated with high levels of negative affect, and with elevated symptoms of anxiety and depression (Fajkowska & Derryberry, 2010; Pacheco-Unguetti et al., 2011; Reinholdt-Dunne et al., 2013, 2009; Tortella-Feliu et al., 2014). There is some evidence that the two components of AC (focusing and shifting) may have specific differential associations with anxiety and depression, such that poor focusing is most strongly correlated with anxiety symptoms, whilst impaired shifting ability is more closely correlated with depressive symptoms (Judah et al., 2014; Reinholdt-Dunne et al., 2013; Ólafsson et al., 2011).

Studies utilising performance measures also indicate consistent evidence that depression is associated with broad impairments in cognitive control, including AC, with deficits apparent in both clinical groups and vulnerable populations (e.g. subclinical depressive symptoms, remitted groups, high trait ruminators) (E. H. W. Koster, Hoorelbeke, Onraedt, Owens, & Derakshan, 2017; Snyder, 2013). This evidence is consistent with the proposal that AC deficits represent a vulnerability factor for affective disorder, rather than resulting only from concurrent affective symptoms.

Relatively few studies have specifically used the ANT to examine the associations between AC and depression and anxiety. Where this has been examined, some studies have found no associations between ANT performance and depression or anxiety (Reinholdt-Dunne et al., 2013). However, others have found reduced executive network performance and changes in orienting function associated with higher anxiety (Pacheco-Unguetti, Acosta, Callejas, & Lupiáñez, 2010; Pacheco-Unguetti et al., 2011). These relationships therefore require further examination and clarification. There is particular debate as to whether the cognitive deficits associated with depression apply to the processing of emotionally neutral information, as presented in the ANT, or whether these deficits are more specific to the processing of affective information. One view is that general deficits may be present for clinical populations, whereas impairments in vulnerable populations may be more restricted to conditions requiring processing of emotional material (E. H. W. Koster et al., 2017; Snyder, 2013). However, this has rarely been systematically examined.

### 1.3 Attentional control and rumination

Emotion regulation strategies are of considerable interest in the context of affective disorder, as they appear to play a role in the development and maintenance of these disorders. For example, use of rumination as an emotional regulation strategy has been strongly related to the development and maintenance of depression (Susan Nolen-Hoeksema, Wisco, & Lyubomirsky, 2008). Rumination is defined as ‘a mode of responding to distress that involves repetitively and passively focusing on the symptoms of distress and the possible causes and consequences of these symptoms' (Nolen-Hoeksema et al., 2008, p. 400). It is understood to be composed of two components – brooding and reflective pondering (Treynor, Gonzalez, & Nolen-Hoeksema, 2003). Reflective pondering involves a purposeful focus on difficulties, in order to facilitate problem solving. In contrast, brooding involves making passive comparisons between one's current situation and some unachieved standard, and is considered to be particularly maladaptive.

It has been suggested that ER strategies in large part rely on the effective use of AC, and therefore deficits in AC may predispose individuals to greater use of maladaptive strategies (Fajkowska & Derryberry, 2010; Joormann & D'Avanzato, 2010; Ernst H. W.; Koster et al., 2011). Several theoretical models particularly implicate AC deficits in tendency to employ high levels of worry and rumination (Hirsch & Mathews, 2012; Ernst H. W.; Koster et al., 2011; Mor & Daches, 2015; Watkins & Nolen-Hoeksema, 2014). Various evidence suggests that worry and rumination are associated with impairments in attention inhibition and shifting, although in common with the literature on depression, there is not clear consensus about whether these impairments apply to neutral as well as affective stimuli (De Lissnyder, Derakshan, De Raedt, & Koster, 2011; Vanderhasselt, Kühn, & De Raedt, 2011; Whitmer & Banich, 2007). One somewhat divergent model has proposed that rumination is associated not with AC deficits as such, but with a narrowed focus of attention (Whitmer & Gotlib, 2013). It is proposed that a narrowed focus of attention could predispose individuals to rumination, by promoting a narrow and persistent focus on negative thoughts. A narrowed focus of attention may confer advantages on some tasks, for example avoiding distraction by irrelevant stimuli, but also confers disadvantages where performance requires attentional flexibility or a broadly distributed focus of attention.

One previous study has specifically examined associations between ANT performance and rumination, finding that poorer functioning of the orienting network explained increases in brooding, even after controlling for trait anxiety (Tortella-Feliu et al., 2014). Several studies have also linked self-reported AC to levels of repetitive negative thinking (RNT), including rumination and worry. These studies consistently indicate that poorer attentional control is associated with higher levels of RNT (Cox, Ebesutani, & Olatunji, 2016; Hsu et al., 2015; Mills et al., 2016).

Researchers have recently started to explore hypothesised three-way relationships between AC, RNT and affective disorder, in particular the suggestion that AC deficits may predispose individuals to depressive symptoms, via an increase in maladaptive ER strategies. Hsu et al. (2015), using path analysis, showed that rumination mediates the relationship between self-reported AC and depression/anxiety symptoms. Similarly, Kertz, Stevens, and Klein (2017) demonstrated a relationship between AC deficits and depression, mediated by an indirect pathway involving RNT and mood recovery. However, these studies rely on cross-sectional data and have used only self-report measures of AC. One previous study has examined the longitudinal relationship between performance on an AC task and later depressive symptoms, showingthat AC deficits at baseline were associated with higher depressive symptoms at 1 year, and this relationship was fully mediated by rumination (Demeyer, De Lissnyder, Koster, & De Raedt, 2012). In addition, cognitive training studies in both clinical and selected vulnerable populations, provide evidence that increasing AC via cognitive training has benefits in terms of reductions in rumination and subsequent depressive symptoms (E. H. W. Koster et al., 2017; Mor & Daches, 2015). These findings are consistent with a model in which AC deficits play a causal role in vulnerability to depressive symptoms. There is then growing support for a model in which AC deficits increase vulnerability to depression, via increases in rumination or other forms of RNT.

### 1.4 Aims

AC impairments appear to be a vulnerability factor for the development of dysfunctional emotion regulation strategies and subsequent affective disorder. This study examined a number of outstanding questions regarding the relationships among attentional performance, self-reported AC, depression, anxiety and rumination in a healthy population. Firstly, the study examined the relationship between attention performance and self-reported AC. Secondly, the study tested whether depression and generalized anxiety symptoms are related to a) attention performance on an affectively neutral task (the ANT), b) self-reported AC. Thirdly, we investigated whether rumination is related to a) attention performance on the ANT, b) self-reported AC. Finally, we tested whether rumination mediates relationships between AC and depression.

It was hypothesised that attentional performance and self-reported AC would be positively, although perhaps weakly, correlated. We also hypothesised that increased symptoms of depression and generalized anxiety would be related to impaired ANT performance, particularly on the conflict index, and that both would be related to impaired self-reported AC. It was further predicted that, in line with previous findings, depressive symptoms would be particularly associated with shifting impairments and generalized anxiety symptoms with focusing impairments. Finally, it was hypothesised that brooding rumination would mediate the relationships between AC impairments and depressive symptoms.

## 2 Method

### 2.1 Participants

81 participants were recruited through a combination of posters, email newsletters and online advertising. In order to ensure a range of depressive symptoms within this non-clinical sample, the study was advertised for participants who had been experiencing recent sadness or low mood, lasting at least 2 weeks. However, participants who were currently receiving or seeking treatment for any psychological disorder not included in the study. One participant was excluded after reporting current treatment for a mental health condition, resulting in a final sample of N = 80. Inclusion criteria included age between 18 and 35 years, normal or corrected-to-normal vision and hearing, and fluent English language. The study was reviewed and approved by the local University research ethics committee (ref. MS-IDREC-C1-2015-038).

### 2.2 Measures

#### 2.2.1 Ruminative Responses Scale (RRS) (S. Nolen-Hoeksema & Morrow, 1991)

The RRS is a 22-item measure of proneness to ruminative thought when feeling sad or low. A total score can be calculated, as well as subscale scores for ‘brooding’ and ‘reflective pondering’ (Treynor et al., 2003). Brooding is conceptualised as involving passive comparisons between one's current situation and some unachieved goal or standard; it has been strongly linked to both current and future depressed mood. In contrast, reflective pondering is understood to involve purposeful engagement in cognitive problem solving.

#### 2.2.2 Attentional Control Scale (ACS) (Derryberry & Reed, 2002)

The ACS is a 20 item self-report measure of the ability to control and direct attention, and to resist distraction. A total score is calculated, as well as two subscale scores for ‘focusing’ and ‘shifting’ attention. Focusing refers to the ability to maintain attention, particularly in the face of distraction. Shifting refers to the ability to direct attention flexibly, and to move attention between different tasks or stimuli. Subscale scores were calculated according to the approach reported by Ólafsson et al. (2011) [see also (Reinholdt-Dunne et al., 2013)].

Patient Health Questionnaire (PHQ-9) (Spitzer, Kroenke, & Williams, 1999) and GAD-7 (Spitzer, Kroenke, Williams, & Lowe, 2006).

The PHQ-9 and GAD-7 are brief questionnaires that assess the presence of symptoms of depression and generalized anxiety disorder over the preceding 2 weeks. These scales have good reliability and validity, and are frequently used as screening tools in both clinical and research settings (Kroenke, Spitzer, & Williams, 2001; Spitzer et al., 2006).

#### 2.2.3 Attentional Network Task (ANT) (Fan et al., 2002)

**Fig. 1:**
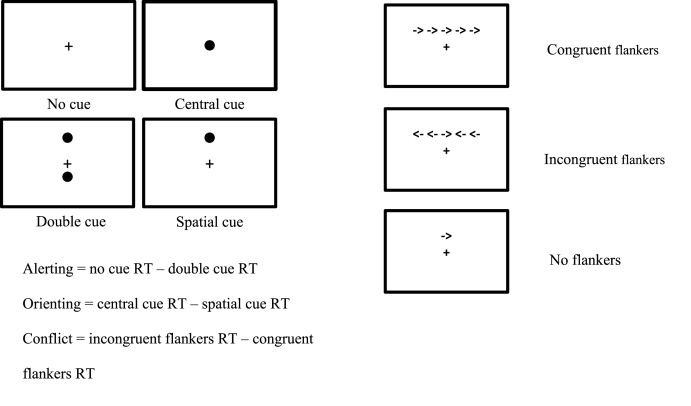
Attentional Network Task displays and index calculations.

The ANT is designed to assess 3 aspects of attentional performance – alerting, orienting and conflict resolution (executive attention). On each trial, participants are asked to indicate the direction of a central arrow, which appears in one of two positions. On some trials the arrow appears with either congruent or incongruent flankers. Target displays are preceded by a cue display with one of 4 configurations: no cues, central cue, double cue, or spatial cue (see Fig. 1).

The task was presented on a laptop, using Inquisit presentation software. Participants completed a practice block (28 trials) during which they received feedback, followed by 2 experimental blocks of 96 trials. Mean reaction times for all trials with a correct response were used to calculate the attention indices, as shown in Fig. 1. Lower values for alerting and orienting indicate poorer performance; for conflict, higher values indicate poorer conflict resolution.

### 2.3 Procedure

Participants attended for a single session of testing and gave written consent to participate. Demographic data and questionnaire measures were completed, followed by the ANT. Following these tasks, participants completed a rumination induction and several other measures, which will be reported elsewhere (Dejong, Fox, & Stein, in preparation).

### 2.4 Statistical analysis

Analysis was conducted using SPSS version 22. Distribution of the data was checked for normality and outliers >3 standard deviations from the mean were removed. This resulted in removal of a small percentage of ANT index scores (0.01%). Pearson's correlations were used to examine relationships between questionnaire measures and ANT performance indices. Hierarchical linear regressions were used to test potential correlates of depression, generalized anxiety and brooding. As differential relationships have previously been shown for depression and anxiety with AC (Judah et al., 2014; Reinholdt-Dunne et al., 2013; Ólafsson et al., 2011), each model included other affective symptoms at Step 1, followed by ACS subscales at Step 2. For example, to examine correlates of depression, generalizes anxiety was entered at Step 1, and ACS subscales at Step 2.

**Fig. 2:**
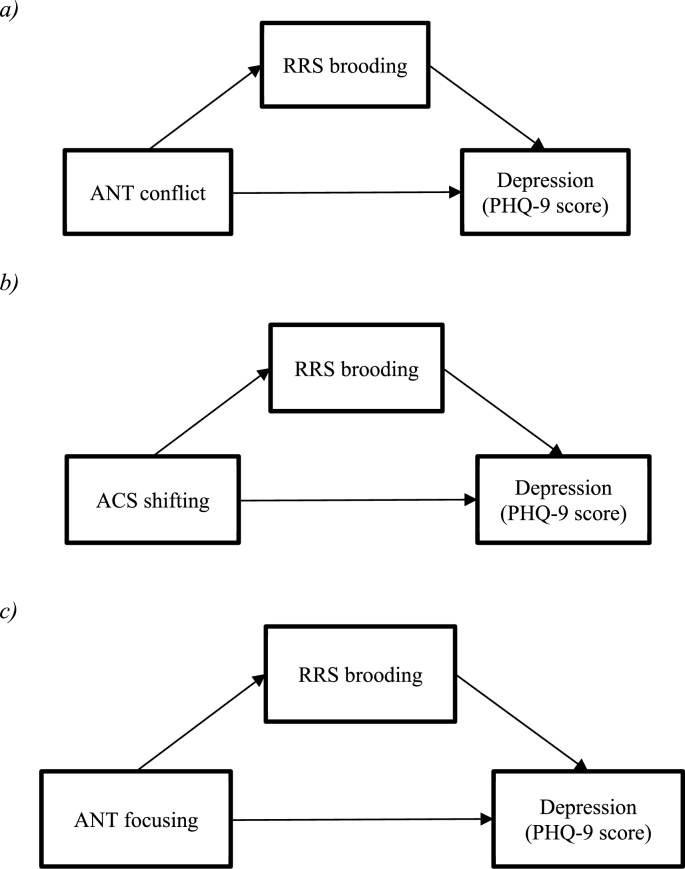
Models examined in the mediation analyses.

Mediation analyses were conducted using the PROCESS macro for SPSS (Hayes, 2013) (processmacro.org). This method assesses both the direct and indirect paths between the independent variable and the dependent variable. In this case, the direct path in each model involved the effects of AC on depression, while the indirect path tested concerned the effects of AC on depression through the hypothesised mediating variable (brooding rumination). These analyses focused specifically on brooding, as this is the aspect of rumination theorised to be most strongly predictive of depression. Analyses were conducted with 10,000 bootstrap samples, and bias-corrected bootstrap confidence intervals are included for all indirect effects. Completely standardized effects are also reported for all indirect effects, as an indicator of effect size. Three models were tested, each including one of the attentional control variables of interest (ACS attentional shifting, ACS attentional focusing, ANT conflict index) as the independent variable, depression score as the dependent variable, and brooding rumination as the hypothesised mediator (see Fig. 2*).*

## 3 Results

### 3.1 Participant characteristics

The sample was composed of 80 participants (27; 33.8% male), with a mean age of 22.82 years (SD = 4.10, range 18–34 years). The ethnicity of the sample was predominantly white (N = 57, 71.3), and the majority of participants were students (N = 58, 72.5%).

### 3.2 Descriptive statistics

Descriptive statistics for questionnaire scores and ANT performance are shown in Table 1. Although there are no established population norms for the ANT, these values appear to be broadly similar to previous studies in undergraduate samples (Reinholdt-Dunne et al., 2013).

**Table 1:** Descriptive statistics for questionnaire measures and Attention Network Task (ANT) performance. PHQ-9 = Patient Health Questionnaire, RRS = Ruminative Responses Scale, ACS = Atttentional Control Scale.

*Measure*	*Mean (SD)*
PHQ-9 score	10.28 (5.55)
GAD-7 score	9.49 (4.46)
RRS Total	48.54 (10.98)
RRS Brooding	11.14 (3.21)
RRS Reflective Pondering	10.81 (3.36)
ACS Total	49.00 (8.73)
ACS Focusing	21.35 (4.59)
ACS Shifting	27.65 (5.34)

	*Mean RT, msec (SD)*
ANT: No cue trials	553.16 (79.37)
Centre cue trials	508.07 (65.11)
Double cue trials	501.17 (61.93)
Spatial cue trials	484.70 (67.12)
Congruent flanker trials	490.85 (69.74)
Incongruent flanker trials	573.74 (75.20)
Alerting	50.36 (29.84)
Orienting	23.46 (20.08)
Conflict	84.55 (31.74)

#### 3.2.1 Correlations between self-report and performance measures of attention

There were no significant correlations between self-reported AC and any of the ANT performance indices – all r's ≤ 0.20, p's > 0.05.

### 3.3 Correlation of attention measures with depression, anxiety and rumination

Depression, generalized anxiety symptoms and rumination were negatively correlated with self-reported AC, such that higher psychopathology was associated with poorer reported AC (see Table 2). This applied for both subscales of the ACS and the total score.

**Table 2:** Correlations between depression, anxiety, rumination and attention measures.

	ACS Total	ACS Focus	ACS Shift	ANT Alerting	ANT Orienting	ANT Conflict
	*r (p)*	*r (p)*	*r (p)*	*r (p)*	*r (p)*	*r (p)*
PHQ-9	−.47***	−.26*	−.54***	−.08	.09	−.28*
GAD-7	−.42***	−.31**	−.42***	−.07	.06	−.25*
RRS Total	−.49***	−.35**	−.49***	−.05	−.11	−.18
RRS Brooding	−.45***	−.38**	−.40***	−.02	−.03	−.29*
RRS Reflective Pondering	−.30**	−.25*	−.28*	−.02	−.32*	.01

ACS = Attentional Control Scale, ANT = Attention Network Task, PHQ-9 = Patient Health Questionnaire, RRS = Ruminative Responses Scale.

***p < .001, **p < .01, *p < .05.

Depression, generalized anxiety and rumination were uncorrelated with the alerting index of the ANT. Only reflective pondering was correlated with orienting, such that higher reflective pondering was associated with poorer orienting. Depression, generalized anxiety and brooding were all negatively correlated with the ANT conflict index. This indicates that higher depression, generalized anxiety and brooding were associated with better conflict performance, i.e. reaction times were less affected by the presence of incongruent flankers (see Table 2).To examine the possibility that effects were driven by trade-offs between speed and accuracy, performance metrics were also examined. Accuracy was high (M = 123.6 of 128 trials correct, SD = 4.3, i.e. 95.6% accuracy) and response accuracy was not correlated with RT for any trial type. Neither was accuracy correlated with depression or generalized anxiety symptoms, or with rumination scores. Associations between depression, anxiety, rumination and ANT index scores were similarly not due to differences in overall RTs on the ANT – there were no correlations between these variables and RT for any trial type (see Table 3, Table 4).

**Table 3:** Regression analysis: correlates of depression (PHQ-9 scores).

	B	SE B	β	*t*	*p*	*95% CI for B*
**Step 1.** △ R^2^ = .515						
GAD-7 score	.902	.100	.718	9.049	<.001**	.70, 1.10
F(1, 77) = 81.881, p < .001, R^2^ = .515, adj. R^2^ = .509						
**Step 2.** △ R^2^ = .080, △ F(2,75) = 7.452, p = .001						
GAD-7 score	.760	.102	.605	7.418	<.001**	.56, .96
ACS focusing	.138	.105	.115	1.315	.193	−.07, .35
ACS shifting	−.359	.094	−.350	−3.824	<.001 **	−.55, −.17
F(3, 75) = 36.835, p < .001, R^2^ = .596, adj. R^2^ = .580						

ACS = Attentional Control Scale.

**Table 4:** Regression analysis: correlates of anxiety (GAD-7 scores).

	B	SE B	β	*t*	*p*	*95% CI for B*
**Step 1.** △ R^2^ = .515						
PHQ-9 score	.571	.063	.718	9.049	<.001**	.45, .70
F(1, 77) = 81.88, p < .001, R^2^ = .515, adj. R^2^ = .509						
**Step 2.** △ R^2^ = .017, △F(2, 75) = 1.336, p = .269						
PHQ-9 score	.557	.075	.700	7.418	<.001**	.41, .71
ACS focusing	−.139	.089	−.146	−1.561	.123	−.32, .04
ACS shifting	.029	.088	.036	.333	.740	−.15, .20
F(3, 75) = 28.422, p < .001, R^2^ = .532, adj. R^2^ = .513						

PHQ = Patient Health Questionnaire, ACS = Attentional Control Scale.

### 3.4 Associations of attentional control with depression and anxiety: regression analyses

In order to examine correlates of depression (PHQ-9 scores), a linear regression model was tested with anxiety (GAD-7 score) entered in the first step, and ACS focusing and shifting entered in the second step. At Step 1, generalized anxiety symptoms were positively associated with depression, accounting for 51.5% of the variance in depression. Adding focusing and shifting to the model significantly improved the model, explaining an additional 8% of the variance. At this stage, both anxiety [β = 0.61, t = 7.42, p < .001] and poorer shifting [β = −0.35, t = −3.82, p < .001] were associated with depression. Focusing was not a significant correlate [β = 0.12, t = 1.32, p = .193].

In order to examine correlates of generalized anxiety symptoms (GAD-7 scores), a model was tested with depression (PHQ-9 score) entered in the first step, and ACS focusing and shifting entered in the second step. This model indicated that at Step 1, depression was significantly correlated with anxiety [β = 0.72, t = 9.05, p < .001], explaining 51.5% of the variance in generalized anxiety symptoms. Adding the ACS subscales at Step 2 did not improve the model fit [△R^2^ = 0.02, F(2, 75) = 1.34, p = .269].

Controlling for age and gender of participants in these models did not alter the findings and so the uncorrected models are reported here.

### 3.5 Associations of attentional control with brooding: regression analysis

In order to examine correlates of brooding, a linear regression model was tested with depression and anxiety scores entered in the first step, and ACS focusing and shifting entered in the second step. At Step 1, depression [β = 0.32, t = 2.77, p = .007] and generalized anxiety [β = 0.46, t = 4.04, p < .001] were both significantly associated with brooding, together explaining 52.1% of the variance. Adding the ACS scores at Step 2 did not significantly improve the model fit [△R^2^ = 0.03, F(2, 74) = 2.21, p = .117].^1^ Controlling for age and gender of participants did not alter these findings, therefore the uncorrected model is reported here (see Table 5).

**Table 5:** Regression analysis: correlates of brooding.

	B	SE B	β	*t*	*p*	*95% CI for B*
Step 1. △ R^2^ = .521						
GAD-7 score	.336	.083	.461	4.043	<.001**	.17, .50
PHQ-9 score	.183	.066	.315	2.765	.007**	.05, .32
F(2, 76) = 41.313, p < .001, R^2^ = .521, adj. R^2^ = .508						
**Step 2.** △ R^2^ = .027, △ F(2, 74) = 2.207, p = .117						
GAD-7 score	.304	.083	.417	3.646	<.001**	.14, .47
PHQ-9 score	.188	.071	.324	2.635	.010**	.05, .33
ACS focusing	−.131	.065	−.189	−2.009	.048*	−.26, −.001
ACS shifting	.027	.063	.046	.433	.667	−.10, .15
F(4, 74) = 22.416, p < .001, R^2^ = .548, adj. R^2^ = .523						

PHQ = Patient Health Questionnaire, ACS = Attentional Control Scale.

### 3.6 Mediation analyses

Firstly, the effect of attentional conflict performance (ANT conflict index) on depression was examined, using single-step mediation with brooding rumination as the potential mediator (Fig. 2a). There was no significant direct effect of conflict performance on depression (B = −0.02, t(77) = −1.11, p = .272, 95%CI [−0.05, 0.01]). However, there was a significant indirect effect via brooding (B = −0.03, 95% CI [−0.05, −0.01]). The completely standardized indirect effect was B = −0.18 (95%CI [−0.29, −0.05]), that is for every 1 sd change in the ANT conflict index, there was a −0.18 sd change in depression score, via the indirect pathway.

Secondly, the effect of attentional shifting on depression was tested, with brooding rumination entered as the potential mediator (Fig. 2b). There was a significant direct effect of attentional shifting on depression (B = −0.34, t(78) = −3.79, p < .001, 95%CI [−0.52, −0.16]). The indirect path was also significant, i.e. brooding rumination significantly mediated the relationship between attentional shifting and depression (B = −.22, 95% CI [−0.36, −0.11]). The completely standardized indirect effect was B = −0.21 (95%CI [−0.33, −0.11]).

Finally, the effect of attentional focusing on depression was examined, with brooding rumination as a potential mediator (Fig. 2c). There was no significant direct effect of attentional focusing on depression (B = −0.02, t(77) = −0.18, p = .856, 95%CI [−0.25, 21]). There was however a significant indirect effect via brooding (B = −0.29, 95% CI [−0.46, −0.13]). The completely standardized indirect effect was B = −0.24 (95%CI [−0.38, −0.11]).

All indirect effects remained significant when age, gender and generalized anxiety symptoms were added to the mediation models as covariates. The uncorrected models are reported here.

## 4 Discussion

This study examined a number of questions regarding the relationships between attentional performance, self-reported AC, depression, anxiety and rumination.

### 4.1 Self-reported attention, depression and rumination

The results indicate that depression, generalized anxiety and rumination are associated with impairments in both the shifting and focusing components of self-reported AC. Regression analyses indicated that, after controlling for generalized anxiety, depression was specifically associated with the shifting subscale of the ACS. This is consistent with previous findings indicating a specific relationship between depression and this component of AC (Judah et al., 2014; Reinholdt-Dunne et al., 2013; Ólafsson et al., 2011). ACS scores did not correlate with generalized anxiety after controlling for depression. Similarly, ACS scores were not associated with brooding after controlling for depression and anxiety. However, the relationships between self-reported AC and depression were mediated by brooding, which is consistent with the hypothesis that AC difficulties confer risk for depression because they predispose individuals to use brooding as an emotional regulation strategy. This increase in brooding, in turn, increases risk for depression (Joormann & D'Avanzato, 2010).

### 4.2 Attentional performance, depression and rumination

Against expectation, depression, anxiety and rumination were associated with better conflict performance on the ANT, i.e. at higher levels of psychopathology performance was *less* affected by the presence of incongruent flankers. A mediation analysis confirmed an indirect association between ANT conflict performance and depression, mediated by brooding.

Enhanced conflict performance associated with depression, anxiety and rumination may appear to be a surprising finding. However, it is consistent with several previous studies indicating that participants high in depression and rumination can demonstrate preserved or even enhanced ability to inhibit or ignore distracting information (Joormann and D'Avanzato, 2010; Krompinger & Simons, 2011; Meiran et al., 2011; Zetsche et al., 2012). As outlined in the introduction, one prominent theoretical model has sought to explain these findings, by proposing that rumination is associated with a narrowed focus of attention (Whitmer & Gotlib, 2013). This has advantages for particular tasks, for example avoiding distraction by irrelevant stimuli, but also confers disadvantages where performance requires attentional flexibility or a broadly distributed focus of attention. It is proposed that a narrowed focus of attention may predispose people to rumination, as it facilitates a narrow and persistent focus on negative thoughts. Habitual rumination is then hypothesised to confer risk for developing depressive symptoms. This model is consistent with the results of the mediation analysis reported here, with the association between ANT conflict performance and depression being mediated by brooding.

### 4.3 Relationships between self-report and performance measures

In this study, self-reported AC and ANT performance were not correlated with one another. This suggests that these measures assess somewhat different constructs. The ANT is a highly controlled test, with simple stimuli, whilst the ACS enquires about performance in complex, everyday situations, which may have rather different attentional demands. In addition, while the ANT assesses several components of attention, it is not considered to be a comprehensive assessment of all aspects of attention performance. The lack of correlations between these measures could also suggest that participants are relatively poor at evaluating their own attention performance, or are influenced by factors other than performance, e.g. personality, mood (Buchanan, 2016; Meltzer et al., 2016; Williams et al., 2017). These findings suggest that self-report and performance measures of attention contribute different information, and there may be benefits to including both types of measure in studies of this kind. The possible reasons for a lack of correspondence between these measures could also be usefully explored in future research, for example by examining additional performance measures, or by manipulating variables that may affect self-assessment of performance.

### 4.4 Strengths, limitations and future directions

Whilst previous work has linked AC to affective disorder and to emotional regulation strategies, relatively few studies have examined the three-way interactions between these constructs. This study therefore helps to extend previous work in this area, and to establish a model for how these variables may be related. The mediation models presented are consistent with previous studies in this area (Demeyer et al., 2012; Hsu et al., 2015; Kertz et al., 2017), and extend earlier work by including both performance and self-reported measures of attention.

There are several limitations of the current study that should be recognised. The sample was primarily composed of university students and, although mean levels of generalized anxiety and depression were in the ‘moderate’ range (Spitzer et al., 1999, 2006), it was a non-clinical sample. It is unclear therefore how these findings would generalise to other populations, including clinical groups. All measures were taken concurrently, and therefore it was not possible to examine causal relationships between variables, or to be confident about the directionality of effects. For theoretical reasons, effects were examined in one direction (i.e. deficits in AC as vulnerability factors for rumination and subsequent depression). However, it is possible that these relationships are in fact bidirectional or mutually reinforcing (Roberts, Watkins, & Wills, 2016). The use of longitudinal and experimental designs in future studies may help to better elucidate these relationships. A relatively large number of analyses were conducted on the dataset, and there is therefore an increased risk of Type 1 errors. It will be important to examine whether these results are replicated in other samples.

As state rumination was not manipulated or measured, we were not able to examine possible interactions between state and trait effects. Whilst the effects of anxiety were considered, there was no measure of worry used in the current study. It is unclear then whether these findings are specific to rumination, or whether they apply more broadly to other forms of RNT. Similarly, other types of emotional regulation (e.g. distraction, reappraisal) were not examined in this study. It is of interest that rumination was associated with enhanced resistance to distraction from incongruent flankers, which we have argued may reflect a narrowed and inflexible focus of attention. This AC profile could be similarly associated with other emotional regulation strategies, for example difficulties using distraction and reappraisal, which require flexible shifting of attention.

### 4.5 Clinical implications

These findings suggest a role for specific AC deficits in predisposing individuals to developing maladaptive emotional regulation styles and subsequent affective disorder. AC deficits may then be a useful marker of vulnerability to affective disorder, and a potential target for preventative interventions. If replicated, the associations found here between ANT conflict and rumination/depressive symptoms may also suggest that enhanced ANT conflict performance is a specific marker of vulnerability to affective disorder.

In addition, there is some evidence that current treatments do not adequately ameliorate either the AC deficits or high levels of rumination associated with depression, which may increase risk of recurrence (E. H. W. Koster et al., 2017). Addressing AC deficits may therefore provide a valuable adjunct to existing treatments. One approach to targeting AC is via cognitive training procedures, such as working memory training. As outlined above, a number of studies have now demonstrated benefits of such interventions, including reductions in rumination and depressed mood, although findings of transfer are mixed (E. H. W. Koster et al., 2017; Mor & Daches, 2015). These interventions then require refinement and optimisation but have significant potential for benefit, both in reducing vulnerability in high risk populations and in increasing treatment efficacy. These designs also provide a robust means of testing hypothesised causal mechanisms, in order to better establish those factors that are implicated in the aetiology and maintenance of depressive symptoms.

## Funding

This work was supported by the Wellcome Trust under Grant 106284/Z/14/Z, the European Research Council under Grant 324176, and the National Institute for Health Research Oxford Health Biomedical Research Centre.

## Disclosure of interest

The authors report no conflicts of interest.

## Footnotes

1 Given the high correlations between depression/anxiety symptoms and between subscales of the ACS, tests for multicollinearity were conducted and examined. However, no indicators of multicollinearity were identified.
